# Recent Development of Biodegradable Occlusion Devices for Intra-Atrial Shunts

**DOI:** 10.31083/j.rcm2505159

**Published:** 2024-05-08

**Authors:** Yi-Fan Li, Ze-Wen Chen, Zhao-Feng Xie, Shu-Shui Wang, Yu-Mei Xie, Zhi-Wei Zhang

**Affiliations:** ^1^Department of Pediatric Cardiology, Guangdong Cardiovascular Institute, Guangdong Provincial People’s Hospital (Guangdong Academy of Medical Sciences), Southern Medical University, Guangdong Provincial Key Laboratory of South China Structural Heart Disease, 510100 Guangzhou, Guangdong, China; ^2^Department of Cardiac Surgery, Guangdong Cardiovascular Institute, Guangdong Provincial People’s Hospital (Guangdong Academy of Medical Sciences), Southern Medical University, Guangdong Provincial Key Laboratory of South China Structural Heart Disease, 510100 Guangzhou, Guangdong, China

**Keywords:** biodegradable, atrial septal defect, patent ovale foramen, occlusion device

## Abstract

Atrial septal defect (ASD) is the third most common type of structural congenital heart defect. Patent foramen ovale (PFO) is an anatomical anomaly in up to 25% of the general population. With the innovation of occlusion devices and improvement of transcatheter techniques, percutaneous closure has become a first-line therapeutic alternative for treatment of ASD and PFO. During the past few decades, the development of biodegradable occlusion devices has become a promising direction for transcatheter closure of ASD/PFO due to their biodegradability and improved biocompatibility. The purpose of this review is to comprehensively summarize biodegradable ASD/PFO occlusion devices, regarding device design, materials, biodegradability, and evaluation of animal or clinical experiments (if available). The current challenges and the research direction for the development of biodegradable occluders for congenital heart defects are also discussed.

## 1. Introduction

Atrial septal defect (ASD) is the third most common type of structural congenital heart disease, accounting for about 10% of clinical congenital heart defects with an estimated incidence of 100 per 100,000 live births [1, 2, 3]. ASD is caused by abnormal formation of the atrial septum, allowing communication between the two atrial chambers. Secundum ASD is the most common type of variant, constituting for 65–70% of all ASDs [2]. If left untreated, hemodynamically significant ASDs will cause a series of clinical symptoms such as right-sided heart failure, atrial arrhythmias, and pulmonary hypertension [4, 5, 6]. With the development of occlusion devices and improvement of interventional techniques, approximately 80% of secundum ASDs are suitable for transcatheter closure using currently available devices [7, 8]. Compared with surgical procedures, transcatheter ASD closure showed a similar safety and efficacy profile, and provided a variety of advantages, including lower lengths of hospital stay, lower rates of post-procedural infection, and fewer peri-procedural complications [9, 10, 11, 12]. Transcatheter closure of ASD has become a first-line therapeutic alternative for eligible patients in many countries regarding its favorable long-term prognosis [13].

Patent foramen ovale (PFO) is valve-like structure space bounded by septum primum and the septum secundum. PFO is an important channel for fetal normal circulation, allowing blood communication from the right to the left atrium. After birth, pressure increase of the left atrium over right atrium results in spontaneous closure of the foramen ovale, pushing the valve of fossa ovalis against the septum secundum [14]. In 20%–25% of the population, the anatomical closure of foramen ovale does not occur, and the PFO remains patent resulting in the existence of transient, interatrial right-to-left shunt when right atrial pressure grows higher than left atrial pressure [15, 16]. PFO may become symptomatic when clots pass from the right atrium into the left atrium, causing embolization of cerebral, coronary, visceral, or peripheral arteries [17]. Nowadays, percutaneous closure of a PFO in adults has become an alternative treatment for secondary prevention of paradoxical embolism, such as PFO-associated stroke [17], and other PFO-related clinical condition such as decompression sickness, migraine, and arterial deoxygenation syndrome [18, 19].

Since King TD *et al*. [20] introduced the first transcatheter ASD closure using a double-umbrella device in 1976, continuous efforts have been devoted to designing a reliable ASD occlusion device with the purpose of realizing a safe, effective, and user-friendly closure system. In the early 1980s, Rashkind [21] introduced the Rashkind device, which was the first commercially used ASD occluder. To date, there are a variety of commercial occlusion devices available for transcatheter closure of ASD/PFO, including Amplatzer ASD/PFO/Cribriform occluder (Abbott Structural Heart, Plymouth, MN, USA) [22, 23, 24, 25, 26, 27], Occlutech Figulla (Flex) occluder (Occlutech, Jena, Germany) [28, 29, 30, 31], Gore Helex/Cardioform septal occluder (W.L. Gore and Associates, Flagstaff, AZ, USA) [32, 33, 34, 35], CardioSEAL/STARFlex (NMT Medical, Boston, MA, USA) [36, 37, 38], Solysafe septal occluder (Swissimplant AG, Solothurn, Switzerland) [39, 40, 41], Cera/CeraFlex occluder (Lifetech, Shenzhen, China) [42, 43, 44], and Cardio-O-Fix occluder (Starway Medical Technology Inc, Beijing, China) [45, 46]. These occlusion devices are mainly composed of elastic memory alloy skeleton (nitinol alloy, cobalt-based alloy, and stainless steel) and biostable membranes [polyethylene terephthalate (PET), e-polytetrafluoroethylene (e-PTFE), polyester, polyvinyl alcohol, and polyurethane membrane, etc.] [47]. Given their good shape-memory performance, these devices can realize excellent closure effect and easy operation process. However, long-term presence of non-degradable metal alloy in the heart may cause a series of potential complications, such as (1) mechanical complications such as erosion, perforation, and pericardial tamponade [48, 49, 50]; (2) atrial arrythmias [51, 52, 53]; (3) nickel allergy [54, 55]; (4) thrombus formation [56, 57]. Furthermore, permanent existence of metal alloy materials will cause obstruction of trans-septal access for potential procedures of left-sided heart diseases, such as left atrial appendage closure, mitral valve repair or replacement, and arrhythmia studies. Although a novel puncturable ASD occluder (ReAces device) consisting of memory nickel-titanium wire and PET membrane has been recently introduced by Zhang X *et al*. [58], it is still in the research stage. Therefore, the evolution of biodegradable implants presents great advantage in the aspect of reducing device-associated short- or long-term complications. The concept of a biodegradable device is that it serves as a temporary scaffold for tissue endothelialization after the defect is closed, and that it will be controllably degraded and “disappear” over time, leaving the new “repair” tissue covering the defect. Hence, maintaining a balance between sufficient tissue endothelialization and a suitable degradation rate of the materials has become a crucial issue for the development of biodegradable occlusion devices.

Recently, biodegradable materials, such as polylactide (PLA), polydioxanone (PDO), polycaprolactone (PCL), polyglycolide (PGA), and poly (lactic-co-glycolic acid) (PLGA), have aroused numerous research interests. In regard of their excellent biocompatibility and bioresorbability, these biodegradable materials have been widely applied in the biomedical field, including implants, coronary stents, drug delivery, tissue engineering, and heart valve [59, 60, 61, 62, 63]. During the past decade, the development of biodegradable occluders has gone through a process from “partially bioabsorbable” to “fully bioabsorbable”. A variety of biodegradable cardiac septal defect occlusion devices, especially ASD/PFO occluders, have been introduced, some of which have showed preliminary favorable outcomes in the human body [14, 64, 65]. This review will address progress made on the innovative design and characteristics of partially and fully biodegradable ASD/PFO occluders, including the materials used in the devices (framework, membranes, and accessory materials), the design and construction of the devices, the results of preclinical and clinical experiments, and the benefits and drawbacks of the occlusion devices. Finally, the current challenges and the research direction of the development of biodegradable occluders are proposed.

## 2. Partially Biodegradable Devices

### 2.1 Biostar and BioTrek Device

**Fig. 1.**
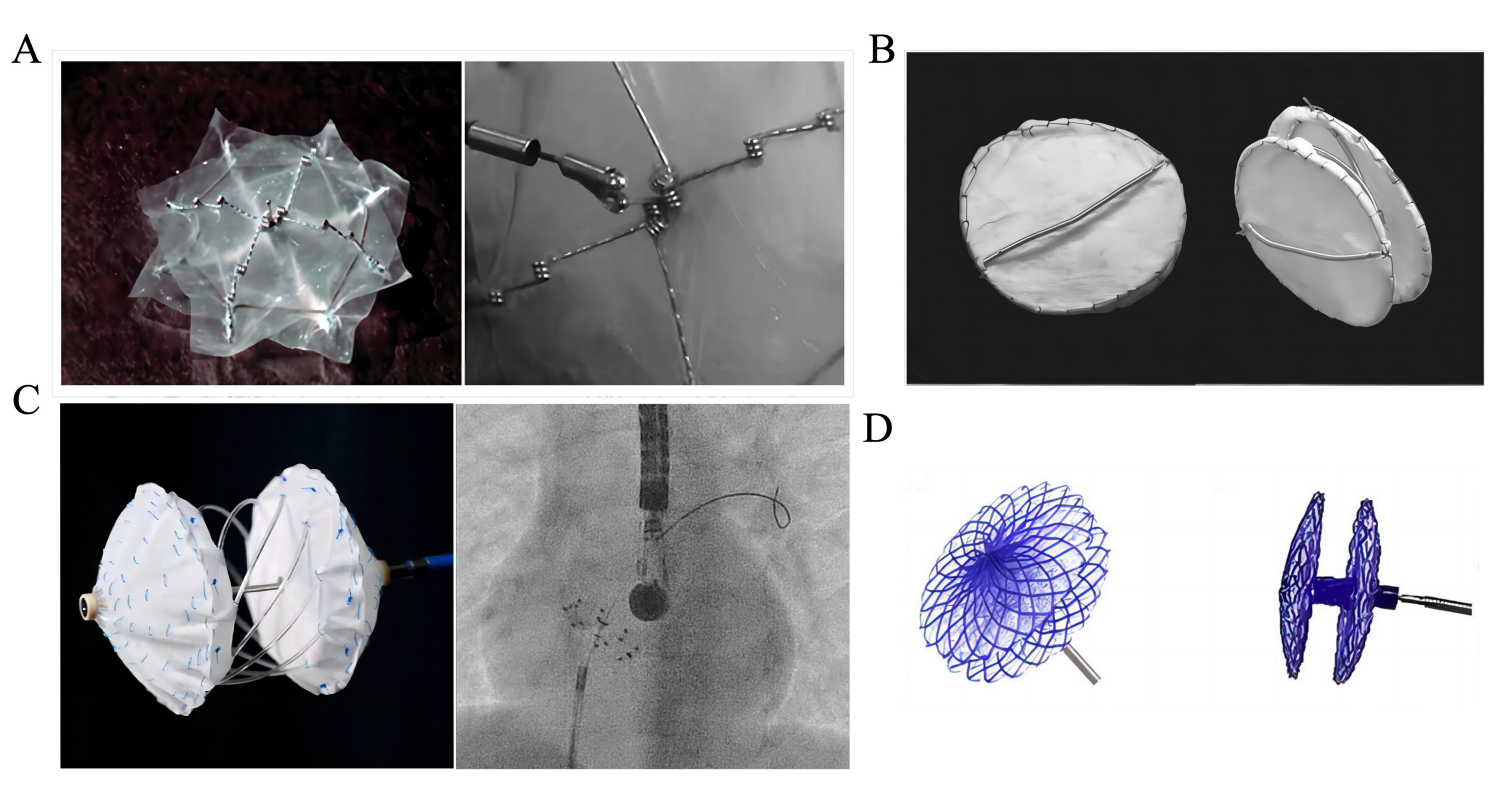
**Partially biodegradable ASD/PFO devices**. (A) The BioSTAR device. Reproduced with permission from [70]. Copyright©2010 John Wiley and Sons. (B) The Double BioDisk. Reproduced with permission from [81]. Copyright©2012 Association of Radiology and Oncology. (C) The Carag Bioresorbable Septal Occluder. Reproduced with permission from [83]. Copyright©2022 Hindawi. (D) The Pancy® occluder. Reproduced with permission from [87]. Copyright©2022 Frontiers. ASD, atrial septal defect; PFO, patent foramen ovale.

The Biostar device (NMT Medical, Boston, MA, USA) is the first partially biodegradable device dedicated for percutaneous ASD/PFO closure in human [66, 67]. It consists of a non-biodegradable MP35N STARFlex (NMT Medical, Boston, MA, USA) framework [68, 69], and a biodegradable membrane consisting of acellular bioengineered type I collagen derived from porcine submucosa (Fig. 1A, Ref. [70]). After device implantation, the collagen membrane is rapidly fused with the atrial septum, and 90–95% of the membrane is reabsorbed and replaced by connective and endothelial tissue over a period of 24 months [66, 71]. The BioSTAR exhibited low immune response with focal mild-to-moderate lymphocyte infiltration, which gradually disappeared once the intestinal collagen layer had completed degradation [66]. In addition, the heparin-coated design could reduce plasma protein and blood cell deposition, thus leading to a decreased thrombogenicity of the device [66].

BioSTAR Evaluation Study was the first clinical trial evaluating efficacy and safety of biodegradable occlusion devices for congenital heart defects [67]. In this study, the BioSTAR device was successfully implanted in 57 (98%) of 58 adult patients diagnosed as ASD or PFO. Successful closure rate was 92% (48/52) at 30 days and 96% (54/56) at 6 months, respectively. The most common adverse event after implantation was transient atrial arrhythmia (8.6%). No evidence of systemic inflammatory response was reported. Based on these encouraging short-term results, the BioSTAR device has been commercially approved in Europe for ASD and PFO closure in 2007 [72, 73, 74, 75], and was used primarily for PFO closure for prevention of recurrent stroke in Canada [70, 76, 77]. Although the BioSTAR device was withdrawn from the market in 2011 because of late complications such as wire fractures and local inflammatory reactions [73], it represented an important breakthrough from non-bioabsorbable metal devices to bioabsorbable devices.

The BioTrek device (NMT Medical, Boston, MA, USA), developed after the Biostar device, is a fully biodegradable device consisting of poly-4-Hydroxybutyrate (P4HB), which causes less inflammatory response and exhibits favorable biocompatibility. BioTrek was reported under preclinical evaluation, but further studies were terminated due to the collapse of NMT Medical in 2011 [78].

### 2.2 Double Biodisk

The Double BioDisk (DBD; Cook Medical, Bloomington, IN, USA) is designed to be a partially biodegradable occlusion device for ASD closure, based on the improvements of Monodisk [79] and BioDisk (a single disk device dedicated for PFO closure) [80]. The DBD consists of two nitinol rings, which are connected with small cannulas and covered with a porcine small intestinal submucosa (SIS) served as a blood flow barrier [81] (Fig. 1B, Ref. [81]). The DBD is a self-expanding and self-centering device, which can be redeployed or retrieved if released inappropriately or lost.

The preclinical study evaluating the efficacy and safety of DBD was conducted in 10 adult sheep ASD models [81]. After percutaneous implantation of DBD, ICE demonstrated complete closure of the defects without residual shunting around the implants, and macroscopic and histologic evaluation showed that DBDs were well incorporated in the atrial septum with complete shunt closure at 6-, 12-, 24- and 52-week follow-up. Furthermore, after implantation, the inflammatory response almost disappeared at 6-month follow-up, and no thrombus formation was detected due to the rapid endothelization process. This study demonstrated that DBD enjoyed favorable efficacy and safety in short and moderate term follow-ups in an adult sheep ASD model.

### 2.3 Carag Bioresorbable Septal Occluder (CSBO)

The Carag Bioresorbable Septal Occluder (CSBO, CARAG AG, Baar, Switzerland) is a self-centring device consisting of a biodegradable PLGA framework with two opposing foldable polyester covers attaching to the framework [82] (Fig. 1C, Ref. [83]). A non-resorbable filament holder made of polyetheretherketone (PEEK) was placed at each end of the filaments. To ensure its X-ray visibility, CSBO contains platinum-iridium markers and a nut made of Phynox (a cobalt-chromium-nickel alloy) at the distal tip of the device [82]. There are 3 sizes of CSBO available for defect closure: type small (to close defects 4 to 12 mm), type medium (to close defects 11 to 20 mm), and type large (to close defects 21 to 25 mm) [83, 84, 85]. The delivery system consists of two coaxial control catheters, which enables the device to be easily configured to a flat double disc shape by independent control of the left and right discs. This implantation technique has been described before for the Solysafe septal occluder [39, 40].

The preclinical study demonstrated that complete endothelialization of CSBO was achieved within 3 months after implantation [82]. The bioresorption of PLGA started after 6 months, and was almost completely degraded by 18–24 months after implantation [84]. A few chronic inflammatory reactions were detected, including lymphocytic infiltration within the neo-endothelial tissue and foreign body giant cells around the polyester [82]. The first-in-human study (ClinicalTrials.gov: NCT01960491) included 17 patients (10 ASDs and 7 PFOs), and the procedural technical success rate was 88.2% (15/17 patients, 9 ASDs and 6 PFOs) [86]. The clinical effective closure rate was 100% in ASD group and 50% in PFO group (2 moderate shunts, and 1 large shunt) at 24-month follow-up, respectively. Based on these successful preliminary results, CSBO achieved CE marking in 2017. Recently, excellent efficacy and safety of CSBO in 4 pediatric patients was demonstrated at 12-month follow-up, with no residual shunts, no device-related complications, no local or systemic inflammatory responses, and no relevant thickness increase of the neo-endothelium within the atrial septum [83].

The CBSO is now the “reSept™ ASD Occluder” (atHeart Medical™ AG, Baar, Switzerland), which is at clinical trial stage in the USA under an investigational device exemption.

### 2.4 Pancy® Occluder

The Pancy® occluder (Shanghai Mallow Medical Instrument Co., Ltd, Shanghai, China) is a partially biodegradable PFO occluder, which is composed of a double-disc PDO framework, interlayer PET membrane, and degradable nylon thread suture [87] (Fig. 1D, Ref. [87]). Preclinical study in beagle dog models showed that the discs could be absorbed within 6 months after implantation *in vivo*, but no detailed animal study results were published [87]. The occluder is currently available in 7 sizes: 18/18, 24/18, 24/24, 30/24, 30/30, 34/24, and 34/34 mm for the right and left atrial discs, respectively. The waist height of the occluder has 3 different sizes (3, 4.5, and 5.5 mm), depending on the variable sizes of the disc.

The multicenter clinical study evaluating the safety and efficacy of the Pancy® occluder in treating PFOs was conducted in Mainland China since 2019 (Clinical Trial Registration: ChiCTR1900024036) [87, 88, 89]. A total of 138 patients were enrolled from 6 medical centers. Several single-center studies results have been published, with successful PFO closure rate ranging from 95.5%–100% at 12-month follow-up. Du Y *et al*. [88] reported that thrombus formation was detected on the surface of the right disc in 3 patients (3/44, 6.8%) at 3- and 6-month follow-up. The thrombi disappeared after 1 month of intensive anticoagulation treatment in these 3 cases. As for the device degradation process, follow-up echocardiography show that degradation of the framework started at 3-month follow-up and was mostly completed at 6-month follow-up in human [89].

## 3. Fully Biodegradable Device

### 3.1 Double-umbrella Occluder

**Fig. 2.**
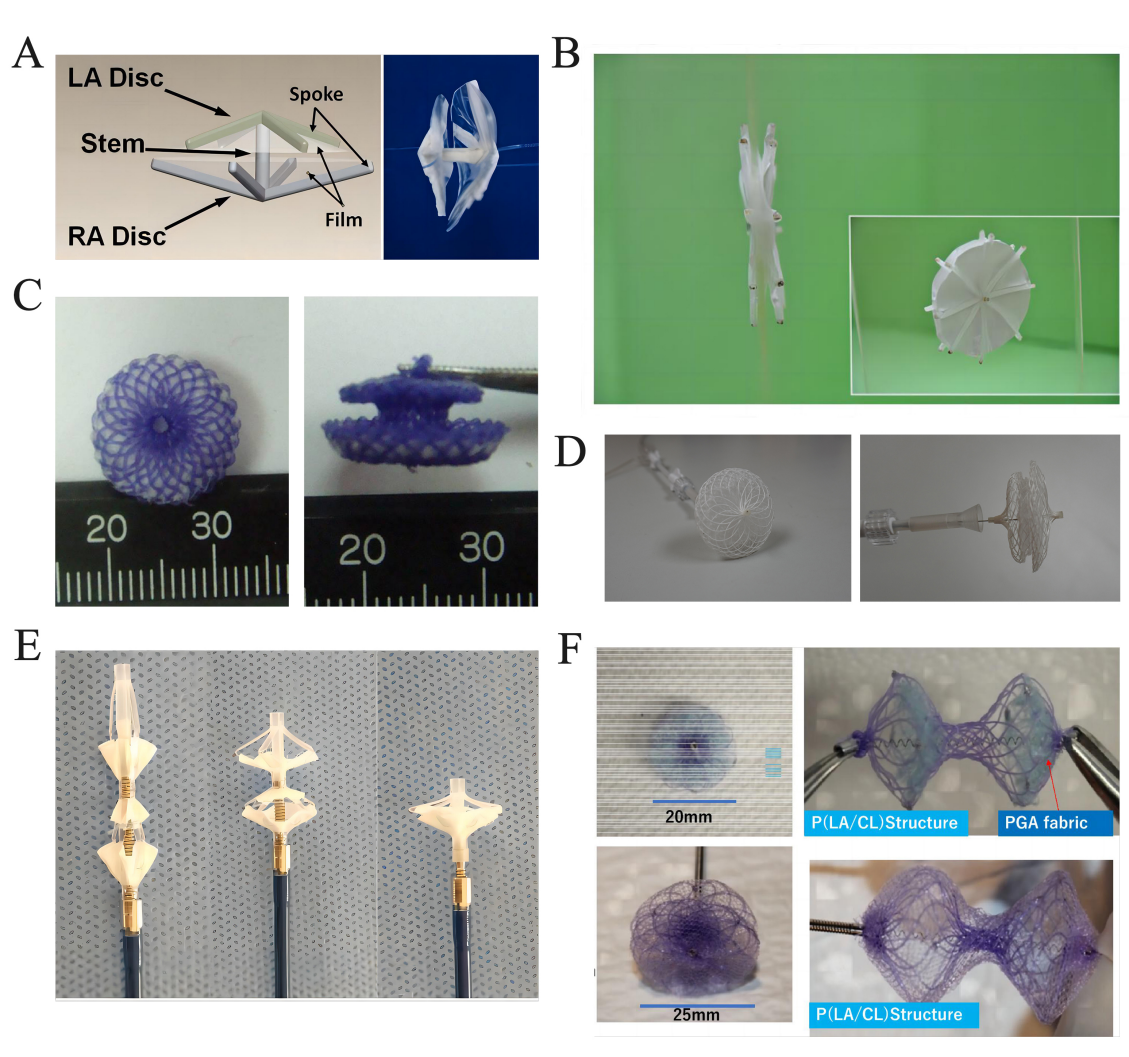
**Fully biodegradable ASD/PFO devices**. (A) The double-umbrella occluder. Reproduced with permission from [90]. Copyright©2010 John Wiley and Sons. (B) The PCL-PLGA/collagen occluder. Reproduced with permission from [92]. Copyright©2011 Springer. (C) The Fully biodegradable ASD occluder. Reproduced with permission from [93]. Copyright©2012 Hindawi. (D) The AbsnowTM PLLA occluder. Reproduced with permission from [96]. Copyright©2021 Hindawi. (E) The PLA-based ASD occluder. Reproduced with permission from [99]. Copyright©2018 John Wiley and Sons. (F) The bioabsorbable ASD/PFO occluder. Reproduced with permission from [104]. Copyright© 2022 Springer. ASD, atrial septal defect; PFO, patent foramen ovale; PCL, polycaprolactone; PLGA, poly (lactic-co-glycolic acid); PLLA, poly-L-lactic acid; LA, left atrial; RA, right atrial; P(LA/CL), poly (L-lactide-co-epsilon-caprolactone); PGA, poly glycolic acid; PLA, poly lactic acid.

The double-umbrella occluder was designed by Duong-Hong D *et al*. [90] in 2010. It is a fully biodegradable device designed for PFO closure, consisting of two self-expanding umbrellas disc made of PCL covered with PLC firms, and eight symmetrically spokes which are made of polylactide-co-ε-caprolactone (PLC) (Fig. 2A, Ref. [90]). The discs are fixed together with a stretchable stem, allowing the left disc anchoring against the interatrial septum wall, while the right disc closing the defect to ensure satisfactory sealing effect [90]. *In-vitro* studies showed that the molecular weight loss of PCL and copolymer PLC were 10% and 20%, while the reduction of storage modulus of PCL and PLC were 15% and 30%, respectively after 12 weeks. The devices were in stable position without residual shunts, and almost complete endothelialization was achieved at 1 month post deployment in two Yorkshire swine. However, moderate thrombus formation and moderate inflammatory responses were seen at 1-month follow-up, suggesting that the biocompatibility and thrombogenic profile of the device should be improved.

### 3.2 Chinese Lantern (CL) Device

The CL device is a fully biodegradable device designed by Venkatraman SS’s group in 2011 [91]. The CL device consists of soft portion (“head”, “waist”, and “tail” films) which are made of a blend of PLC and ${\mathrm{BaSO}}_{4}$, and structural skeleton (lock, head tubes, and wires) which are made of a blend of PCL and ${\mathrm{BaSO}}_{4}$. The X-ray visibility and improved device mechanical properties can be realized by the addition of ${\mathrm{BaSO}}_{4}$ [65]. Folding and sealing of the CL device can be achieved with a novel pull-fold mechanism. Upon retraction of the loop wire, the head films and the tail films would be fold into the working structure [91]. In addition, the length of the waist film could be adjusted to adapt to the native morphology of the atrial septum [91].

The CL devices were successfully implanted percutaneously in two Yorkshire swine ASD/PFO models. 1-month follow-up demonstrated that the devices were in stable position without residual shunting, and complete endothelialization was observed. No apparent thrombi were observed on the device surface, and only mild infiltration of inflammatory cells around the device was seen in histologic examination. However, this version of CL device could not produce sufficient anchorability and enough coverage. Therefore, the sealing effect of the CL device may be suboptimal for larger defects. A new version of CL device has been developed to resolve the shortages of the current design, but no further studies have been reported.

### 3.3 PCL-PLGA/Collagen Occluder

The PCL-PLGA/collagen occluder is a novel biodegradable ASD occlusion device consisting of PCL skeleton fabricated by micro-injection molding and PLGA/collagen nanofibrous membranes using electrospinning techniques [92] (Fig. 2B, Ref. [92]). *In vitro* studies showed that the PCL occluder exhibited comparable compression resistance to that of the Amplatzer ASD occluder. Furthermore, the PCL-PLGA/collagen occluder showed superior sealing capability to that of the Amplatzer occluder. In addition, nanofibrous PLGA/collagen membranes enjoyed excellent capacity in promoting cell proliferation. Nevertheless, no *in vivo* studies of the PCL-PLGA/collagen occluder have been published as yet.

### 3.4 Fully Biodegradable ASD Occluder

A fully biodegradable ASD occluder, namely the improved Amplatzer occluder, was produced in 2012 [93]. The occluder design is similar to the that of the Amplatzer ASD occluder. It is a self-expandable double-disc device, which is composed of a skeleton made of 0.298 mm PDO monofilaments and PLA membranes filled with both discs (Fig. 2C, Ref. [93]). Two tantalum particles are fixed at the edge of each disc as markers under fluoroscopy. Owing to the good elastic property of PDO, the device can be compressed radically, facilitating transcatheter delivery and release.

The fully biodegradable ASD devices were deployed percutaneously in 16 canine ASD models. Animal studies showed that device was completed covered with endothelial cells at 12-week follow-up, and that the PDO framework was mostly degraded and replaced by endogenous host tissue at 24-week follow-up. Histopathological examination showed that significant inflammatory responses were presented at 8 weeks after procedure, and completely disappeared at 24 weeks. The fully biodegradable ASD occluder demonstrated initial promising results with a high procedural success rate, low complication rate, and excellent degradability. However, caution should be observed during long-term follow-up, as the PLA needs at least 2 years to dissolve *in vivo*. Besides, the relatively large diameter of the PDO monofilament (0.298 mm) leads to difficulties in preparation of larger size occluders. Further efforts are needed to improve the design and properties of the device.

### 3.5 ${\mathrm{Absnow}}^{\text{TM}}$ poly-L-lactic acid (PLLA) Occluder

The ${\mathrm{Absnow}}^{\text{TM}}$ PLLA occluder (Lifetech Scientific, Shenzhen, China) is a novel, total bioabsorbale device dedicated for transcatheter ASD closure [94]. The double-disc skeleton is made of 0.15 mm PLLA wire meshes, bonded with three pieces of PLLA membrane at both discs and the waist [95] (Fig. 2D, Ref. [96]). A locking system, consisting of a PLLA locking piece and a controlling handle, is designed to realize sufficient shaping and locking of the device. By controlling the button on the handle, the device can be at the “unlocked” or “locked” state [97]. Seven radio-opaque marks made of platinum-iridium are added on its framework to enhance X-ray visibility. The device is available in waist sizes from 6 to 32 mm at 2-mm increment [96].

In animal studies, 44 PLLA occluders were implanted percutaneously in swine ASD models. Follow-up results demonstrated that the PLLA occluders were completely endothelialized at 3 months [95], and were almost degraded after 36 months of implantation [97]. Compared to nitinol device, the PLLA occluder exhibited more significant local inflammatory reaction within 1 year [95]. Nevertheless, 3-year follow-up in animal study showed very few inflammatory responses whereas the PLLA occluder was almost completely degraded [97]. Based on these promising preclinical results, the first-in-human study was conducted in 5 pediatric patients with moderate or large size ASDs in 2018 [97, 98]. The PLLA occluder presented excellent short-term safety and efficacy in human without complications or significant residual shunts [97]. Although 3-year follow-up results showed that the PLLA device enjoyed favorable safety profile, the high rate of residual shunts (1 large residual shunt and 2 moderate residual shunts) suggested that the device efficacy was suboptimal [96]. A multicenter clinical trial (NCT03601039) has been conducted in August 2018 in Mainland China to further evaluate the effectiveness and safety of the PLLA occluder on ASD closure.

### 3.6 Memosorb® PFO Occluder

The Memosorb® PFO occluder (Shanghai Shape Memory Alloy Co., Ltd, Shanghai, China), is a fully biodegradable device evolved from the PLA-based ASD occlude [99]. The PLA-based ASD occluder is composed of a PLLA skeleton, PLLA locking tube, and two PDLLA discs (Fig. 2E, Ref. [99]). The device can be transformed to tube-like for delivery or umbrella-like for defect closure by controlling its novel delivery system, which consists of an external pushing tube and an internal wire rope. A locking system, which connects the skeleton and the pushing tube, enables locking of the device at different states. In addition, unlike the traditional “waist”, the two discs were connected by the pentagonal skeleton, so the device can be more suitable for defects with narrow paths such as multi-fenestrated ASD and PFO.

Animal studies of the PLA-based ASD occluder were carried out in 18 sheep ASD models. No residual shunt was detected, and no procedure-or device-related complications were noted. The occluders were fused with host native tissue at 1 year, and the molecular weight of the framework decreased to 9% of initial at 2 years. However, the degradation process was not completed at 2 years due to the low degradation velocity of PLLA. In addition, a mild inflammatory response was seen with lymphocytic infiltration around the PLLA skeleton at 2-year follow-up, indicating that the healing response was still underway.

Thus, the second generation occluder, the Memosorb® PFO occluder was designed to improve the degradation and mechanical properties of the device. The concept of its design is in accordance with that of Memosorb® VSD device [100], which is composed of a double-disc framework made of PDO monofilament with PLLA membranes filled in both discs. However, no detailed information about the structure of this PFO occluder has been reported. Compared to the PLA-based occluder, the PDO framework provides superior supporting strength and faster degradation process after endothelization. Moreover, preclinical studies confirmed that the PDO framework had been fully covered with endothelial cells at 6 months. Meanwhile, the device could still retain framework integrity, while the PDO framework was completely degraded at 24 months. Histopathological examination demonstrated that no significant inflammatory response, no thrombus formation, and no myocardial necrosis occurred in the heart. These results indicated that PDO could maintain a stable scaffold for endothelial adhesion before degradation process initiates [101]. A multicenter, randomized, controlled trial (NCT03941691) has been undertaken to validate the safety and reliability of Memosorb® VSD occluder. It has been proved that this fully biodegradable VSD occluder presented similar efficacy and safety compared to that of a commercial metal occluder at 24-month follow-up [100, 102, 103]. The only device-related complication was cardiac arrhythmia, with an incidence of 5.56% for the biodegradable VSD occluder. Moreover, the incidence of sustained conduction block was significantly lower in biodegradable occluder group than in metal device group (*p* = 0.036) [100]. A clinical trial of the Memosorb® PFO occluder has been conducted in Mainland China, however data remains limited.

### 3.7 Bioabsorbable ASD/PFO Occluder (BAO)

Recently, the BAO is introduced by Shinoka T’s team [104]. The 1st generation BAO is made from both 4-0 Poly (l-lactide-co-ε-caprolactone) (PLCL) and 15.2 µm PGA biodegradable polymers. The device has a symmetric double-disc framework, aiming to close PFOs and ASDs. The diameter of left/right disc is 20 mm/15 mm, and the diameter of the central connecting waist is 5 mm (Fig. 2F, Ref. [104]). A 0.9 mm nitinol spring is fixed in the central axis to realize its X ray visibility. After the BAO was successfully implanted percutaneously in 3 out of 4 sheep PFO models, the devices were found did not adequately affix to the atrial septum, while the polymer material was completely degraded at 1-year follow-up. As a result, in the 2nd generation BAO, some modifications were made, including removal of the PGA fiber, increased thickness of the PLCL fibers, addition of a tubular knit which was made of 7-0 PLCL to form a 2-layer structure to facilitate the endothelialization process, and adjustment of the length of central waist (from 5 mm to 7 mm). Animal studies showed that the 2nd generation BAO exhibited better conformation to the atrial septum than the 1st generation occluder, but slower degradation rate due the device modification. At 1-year follow up, the 2nd generation BAO was fully covered with endothelial tissues, and a mild inflammatory response was indicated. Future preclinical studies are needed to further investigate its effectiveness and safety before human studies.

## 4. Limitations and Future Directions

Table 1 (Ref. [66, 67, 70, 72, 73, 74, 75, 76, 77, 78, 81, 82, 83, 86, 87, 88, 89, 90, 91, 92, 93, 94, 95, 96, 97, 98, 99, 100, 104]) depicts a variety of partially or fully biodegradable ASD/PFO occluders reported in the literature. Although most of the biodegradable devices showed promising short-term efficacy and safety in animal studies or in clinical trials, previous studies demonstrated that one of the drawbacks of biodegradable occluders was the suboptimal closure effect due to the occurrence of residual shunts after the degradation process initiates [73, 74]. Compared to metal devices, biodegradable occluders are more likely to exhibit inferior long-term efficacy in ASD closure [96]. The mechanism of undesirable device efficacy may be related to two aspects: the mechanical properties of the biodegradable materials, and the design of the occluders.

**Table 1.** **Partially or fully biodegradable ASD/PFO devices**.

Occluder	Year of introduction	Application	Biodegadability	Framework	Membrane	Features	Device size	Status	Institution	References
BioSTAR	2006	ASD/PFO	Partially biodegradable	MP35N	Heparin-coated porcine intestinal type I collagen	(1) A nondegradable nitinol MP35N “double umbrella” framework with a porcine intestinal collagen layer as biodegradable membrane. (2) 90–95% of the implant will be absorbed after implantation. (3) Withdrawn from the market because of late complications caused by the framework and the membrane.	23 mm, 28 mm, 33 mm	CE mark; discontinued in 2011	NMT Medical, Boston, MA, USA	[66, 67, 70, 72, 73, 74, 75, 76, 77]
BioTrek	2010	ASD/PFO	Fully biodegradable	P4HB	P4HB	Evolved from the Biostar device	NA	Preclinical testing stage; discontinued in 2011	NMT Medical, Boston, MA, USA	[78]
Double Biodisk	2010	ASD/PFO	Partially biodegradable	Two nitinol rings covered with platinum coil	Porcine small intestinal submucosa	(1) Excellent sealing effect. (2) Could be reimplanted or recaptured in the heart.	18 mm, 23 mm, 28 mm	Animal studies	Cook Medical, Bloomington, USA	[81]
Double-umbrella occluder	2010	PFO	Fully biodegradable	PCL (LA disc and RA disc); PLC (the spokes of the RA disc)	PLC	Two self-expanding umbrellas disc linked by the stem.	NA	Animal studies	Nanyang Technological University, Singapore	[90]
Chinese Lantern device	2011	ASD/PFO	Fully biodegradable	PCL and PLC	PLC	(1) Consists of a soft portion (“head”, “waist”, and “tail” films) and structural skeleton (lock, head tubes, and wires). (2) Uses a unique pull-fold mechanism to realize device shaping, which allowed device repositioned and retrieved.	NA	Animal studies	Nanyang Technological University, Singapore	[91]
PCL-PLGA/collagen occluder	2011	ASD	Fully biodegradable	PCL	PLGA/type I collagen	A double umbrella-like device combined with PCL framework and PLGA/type I collagen nanofibrous membranes	NA	*In vitro* studies	Chang Gung University, Taiwan	[92]
Fully bio-degradable ASD occluder	2012	ASD	Fully biodegradable	PDO	PLLA	(1) A self-expandable double-disc device. (2) Two tantalum particles were placed at the edge of each disc to render its radiopacity.	NA	Animal studies	Second Military Medical University, China	[93]
Carag Bio-resorbable Septal Occluder (${\mathrm{reSept}}^{\text{TM}}$ ASD Occluder)	2014	ASD/PFO	Partially biodegradable	PLGA	Polyester	(1) A self-centring device with a framework consisting of a PLGA monofilament covered with two pieces of polyester. (2) A filament holder which is made of PEEK is placed at each end of the filaments.	Type S: 26 mm, Type M: 28 mm	CE mark; Clinical trial stage in the USA	Carag AG, Baar, Switzerland (atHeart Medical™ AG, Baar, Switzerland)	[82, 83, 86]
${\mathrm{Absnow}}^{\text{TM}}$ PLLA occluder	2016	ASD	Fully biodegradable	PLLA	PLLA	(1) A self-expandable, double-disc structure. (2) Both the framework and the membranes are made of PLLA. (3) Can be “locked” and “unlocked” by a locking system.	6–32 mm at 2-mm increment	Clinical trial stage	Lifetech Scientific, Shenzhen, China	[94, 95, 96, 97, 98]
Memosorb PFO occluder	2018	PFO	Fully biodegradable	PLLA (1st generation) PDO (2nd generation)	PDLLA (1st generation) PLLA (2nd generation)	(1) 1st generation: consists of a PLLA skeleton, PLLA locking tube, and two discs made of PDLLA fabrics. (2) 2nd generation: consists of a double- umbrella PDO framework with PLLA membranes filled in both disks.	5–16 mm	1st generation: animal studies, 2nd generation: clinical trial stage	Shanghai Shape Memory Alloy Co., Ltd (Lepu Medical, Beijing, China)	[99, 100]
Pancy® occluder	2019	PFO	Partially biodegradable	PDO	PET	The device has a double-disc framework which was made of PDO filaments and filled with PET nonwoven fabric at each disc.	18/18 mm	Clinical trial stage	Shanghai Mallow Medical Instrument Co., Ltd, Shanghai, China	[87, 88, 89]
							24/18 mm			
							24/24 mm			
							30/24 mm			
							30/30 mm			
							30/34 mm			
							34/34 mm			
Bio-absorbable ASD occlude (BAO)	2022	ASD/PFO	Fully biodegradable	PLCL/PGA (1st generation) PLCL (2nd generation)	NA	(1) Symmetric double-disc design. (2) A 2-layer structure made from PLCL fibers to promote endothelialization process. (3) The central connecting waist was 5 mm in diameter and 7 mm in length.	5 mm (waist); 25 mm (discs)	Animal studies	Nationwide Children’s Hospital, Columbus, OH, USA	[104]

P4HB, poly-4-hydroxybutyrate; PCL, polycaprolactone; PLC, polylactide-co-ε-caprolactone; PDO, polydioxanone; PLLA, poly-L-lactic acid; PLGA, poly (lactic-co-glycolic acid); PEEK, polyetheretherketone; PDLLA, poly-D L-lactic acid; PET, polyethyleneterephthalate; PLCL, poly-L-lactide-co-ε-caprolactone; PGA, poly glycolic acid; ASD, atrial septal defect; PFO, patent foramen ovale; MP35N, nickel-cobalt-chromium-molybdenum alloy; CE, conformité européene; NA, not applicable; BAO, Bioabsorbable ASD/PFO Occluder.

The main challenge for the biodegradable materials is that an ideal degradation time for defect closure in the human heart is unknown [85]. As for biodegradable occluders, initiation of degradation process before sufficient tissue healing could lead to structure collapse, device fragmentation, and thrombus formation. However, a prolonged degradation process would cause undesirable tissue inflammatory, which would become an obstacle to the formation of healthy neo-tissues. These limitations not only lead to the occurrence of residual shunts, but also have potential influences on the rate of complications, such as thromboembolization, cardiac arrhythmias, and myocardial scar formation. Future effort should be addressed on the balance between degradation process and tissue healing response. In addition, previous device designs were only suitable for secundum ASDs with small to moderate size. Improvements of future devices are needed for sufficient closure of ASDs with deficient rims and large size defects. As for PFO closure, one of the important factors that affect prognosis after percutaneous PFO closure is the absence of residual shunt [105, 106]. Previous studies suggested that residual shunt was present in up to 25% of patients who underwent percutaneous PFO closure using metal devices [107, 108]. Recent clinical studies reported that percutaneous PFO closure with Pancy® occluder exhibited excellent closure effect, with complete closure rate 95.5%–100% [87, 88]. Therefore, the evolution of biodegradable PFO occluder might be a new strategy to reduce the risk of residual shunt after PFO closure. Several possible aspects for future improvement of the biodegradable ASD/PFO occluders are presented.

### 4.1 Design of Locking Components

In order to achieve satisfactory sealing effects, both discs of the ASD/PFO devices should well affix to the atrial septum to accomplish complete endothelialization. However, the elastic recovery performance of biodegradable polymer materials is much weaker than that of traditional alloy materials [64]. This characteristic leads to a reduction of device reliability and stability after deployment. Therefore, a locking system is needed to help fix both the atrial discs together to realize optimal shaping of a biodegradable device. An ideal locking system requires safe and simple qualities including simple operation, straightforward delivery technique, and delayed degradation after endothelialization [64, 109]. Several conceivable structural designs for the locking system have been applied to improve the sealing effect of the device, such as the combination of a locking piece with an angle tip and internal screw and a controlling handle [97], the use of a deployment wire to realize “pull-fold” mechanism [91], a shape line tied on the center of the left disc and a knot on the other end to facilitate framework shaping and provide recover support strength of the device [100]. Previous studies [96, 97, 98, 100, 101, 102] in animals or humans demonstrated that the design of locking components has been a feasible strategy to achieve better conformation of both discs to the atrial septum. Further improvements are required to design a locking system allowing for greater flexibility during deployment and a small learning curve with less difficulties to understand the subtleties of its construction.

### 4.2 Use of Biodegradable Shape Memory Polymers

Biodegradable shape memory polymers (BSMPs) emerged as an appealing option in recent years because of their unique benefits of excellent shape memory performance, tunable materials properties, and potential for bioabsorbable. They can change their shape from a temporary shape to a permanent shape triggered by external stimuli such as temperature (heating or cooling) [110, 111, 112, 113], chemical (water and pH value) [114, 115, 116], and light [117, 118]. Most BSMPs are thermo-responsive materials. Therefore, by using a specific stimulus, such as body temperature, BSMPs can realize automatic switch to desirable shape by inducing their shape memory effect when implanted *in vivo * [111].

With their excellent biocompatibility, BSMPs enjoy considerable potential in medical applications [119]. BSMPs-based polyesters, such as PCL, PLA, PLGA, and their copolymers have been used in research and development of implants, such as stents [111, 120, 121, 122], intravascular plugs [114, 115], wound healing [123, 124, 125], drug delivery vehicles [126, 127], and tissue engineering [128, 129, 130, 131, 132]. Wong YS *et al*. [114] introduced a biodegradable shape memory embolization plug which was consists of a composite of a radio-opaque filler and a PLGA blend coated with a crosslinked poly (ethylene glycol) diacrylate (PEGDA) hydrogel. Before implantation, this thermal and water-triggered BMSP was thermally programmed into a temporary shape. Upon delivered into the vessel, this plug will switch to its permanent shape to realize mechanical occlusion effect triggered by body fluid and body temperature. Animal study demonstrated that complete occlusion effect of the plug was achieved within 2 minutes of implantation in rabbit peripheral arteries. Based on these promising results, it may be a new opportunity to develop BSMP-based cardiac septal defect occluders triggered by body temperature or body fluid.

### 4.3 3D/4D Printed Implantable Devices

One of the drawbacks of biodegradable septal defect occluders that leads to complications such as residual shunts, device embolization, valvular damage was the limited specification of device size and morphology. The three-dimensional (3D)/four-dimensional (4D) printing technology using BSMPs emerges as a promising option for the innovation of next-generation heart defect occluders, owing to its advantages of rapid prototyping, adaptive and controllable designing, and personalized customization, which can effectively recapitulate both the native physiochemical and biomechanical characteristics of the cardiac defect structure [133, 134, 135, 136, 137, 138, 139, 140, 141]. Jia H *et al*. [135] reported a self-expandable, biodegradable shape memory PLA vascular stent prepared by 3D printing. The printed PLA stent was programmed into temporary shape for storage at room temperature. After being implanted, the compressed stent could recover to its original shape by heating. Furthermore, Lin C *et al*. [133] developed a shape memory PLA stent with Negative Poisson’s ratio structure by using 4D-printing. Excellent shape memory behaviors of the PLA stents were demonstrated as *in vitro* feasibility tests showed that the stents can expand the simulated narrow blood vessel rapidly. Sun Y *et al*. [140, 141] introduced a novel 3D-printing biodegradable occluder for cardiac defect using self-developed lactide-glycolide-1,3-trimethylene carbonate (LA-GA-TMC). The occluder was double-disk dumbbell with a central, cylindrical waist with 2 mm in length and 4–10 mm in diameter. *In vitro* study showed that this 3D printing biodegradable device had favorable ductility, recoverability, and compatibility. *In vivo* study in rabbits demonstrated that it presented better biocompatibility than the traditional nitinol alloy and PLLA. Recently, Lin C *et al*. [142] developed a biodegradable, dynamic reconfigurable 4D printed customized bionic VSD occluder, which was made of shape memory polyethylene glycol (PEG)/PLA biocomposites with capability of shape transformation at near body temperature. In order to realize its visuality under X-ray, ${\mathrm{BaSO}}_{4}$ radiopaque fillers were introduced into the PEG/PLA matrix. *In vitro* and *in vivo* studies showed that this 4D printed VSD occluder could realize good mechanical properties, favorable biocompatibility, excellent shape memory performance, and radiopacity. In summary, the combination of BSMPs with 3D/4D printing technology offers a future solution for the innovation of occlusion device for congenital heart defects.

## 5. Conclusions

The clinical application of biodegradable occluders is expected to be a future perspective in percutaneous ASD/PFO closure in terms of their ability of facilitating cardiac tissue regeneration, reducing metal-specific complications, and potential for trans-septal access procedures. Some of the biodegradable ASD/PFO devices have been introduced to the market recently. The realization of eliminating the existence of metal alloy inside the heart will enable biodegradable occlusion devices replace metal devices in certain group of patients, such as children and patients with nickel allergy. The development of biodegradable occluders is attractive, nevertheless, its clinical application remains a long way to go, and more efforts should be dedicated focusing on improving device long-term efficacy and safety. The use of new materials and technologies, such as BSMPs and rapid prototyping technology (3D/4D printing), as well as more subtle and reliable design for device locking components, might be feasible strategies to be applied to create next generation biodegradable occlusion devices in the coming decade.
